# Metabolic Adaptations to Aerobic Exercise in Aged Mice

**DOI:** 10.1093/geroni/igab046.2046

**Published:** 2021-12-17

**Authors:** Tyler Marx, Anastasiia Vasileva, Stephen Hutchison, Jennifer Stern

**Affiliations:** University of Arizona, Tucson, Arizona, United States

## Abstract

Aerobic exercise training is a potent intervention for the treatment and prevention of age-related disease, such as heart disease, obesity, and Type 2 Diabetes. Insulin resistance, a hallmark of Type 2 Diabetes, is reversed in response to aerobic exercise training. However, the effect of aerobic exercise training on glucagon sensitivity is unclear. Glucagon signaling at the liver promotes fatty acid oxidation, inhibits De novo lipogenesis, and activates AMP Kinase, a key mediator of healthy aging. Like humans, aging in mice age leads to a decline in physical and metabolic function. To understand the role of glucagon signaling in exercise-induced improvements in physical and metabolic function in the mouse, we implemented a 16-week aerobic exercise training protocol in young and aged mice. 16 weeks of exercise training initiated at 6 months of age increased markers of physical function (P<0.01) and attenuated age-related weight gain (P<0.05) and fat mass (P<0.0001). Additionally, exercise training improved glucose clearance (P<0.01), enhanced glucose-stimulated insulin secretion (P<0.01) and decreased hepatic lipid accumulation (P<0.05). Importantly, exercise training decreased hypoglycemia stimulated glucagon secretion (P<0.01), with no effect on hepatic glucagon receptor mRNA expression or serum glucagon. Thus, we propose that aerobic exercise training enhances glucagon sensitivity at the liver, implicating glucagon as a potential mediator of exercise-induced improvements in aging. Studies initiating the same aerobic exercise training intervention at 18 months of age in the mouse are currently underway to establish the role of glucagon receptor signaling in exercise-induced improvements in aging.

